# Cell Wall Amine Oxidases: New Players in Root Xylem Differentiation under Stress Conditions

**DOI:** 10.3390/plants4030489

**Published:** 2015-07-14

**Authors:** Sandip A. Ghuge, Alessandra Tisi, Andrea Carucci, Renato A. Rodrigues-Pousada, Stefano Franchi, Paraskevi Tavladoraki, Riccardo Angelini, Alessandra Cona

**Affiliations:** 1 Institute of Crystallography, Consiglio Nazionale delle Ricerche (CNR), Monterotondo 00015, Italy; E-Mail: sandip.ghuge.biotech@gmail.com; 2 Department of Sciences, Università Roma Tre, Roma 00146, Italy; E-Mails: ale1.ale2@alice.it (A.T.); andrea.carucci@uniroma3.it (A.C.); stefano.franchi@uniroma3.it (S.F.); paraskevi.tavladoraki@uniroma3.it (P.T.); riccardo.angelini@uniroma3.it (R.A.); 3 Department of Life, Health, and Environmental Sciences, Università dell’Aquila, L’Aquila 67100, Italy; E-Mail: pousada@univaq.it; 4 Istituto Nazionale Biostrutture e Biosistemi (INBB), Rome 00136, Italy

**Keywords:** amine oxidases, polyamines, hydrogen peroxide, xylem differentiation, cell wall, root

## Abstract

Polyamines (PAs) are aliphatic polycations present in all living organisms. A growing body of evidence reveals their involvement as regulators in a variety of physiological and pathological events. They are oxidatively deaminated by amine oxidases (AOs), including copper amine oxidases (CuAOs) and flavin adenine dinucleotide (FAD)-dependent polyamine oxidases (PAOs). The biologically-active hydrogen peroxide (H_2_O_2_) is a shared compound in all of the AO-catalyzed reactions, and it has been reported to play important roles in PA-mediated developmental and stress-induced processes. In particular, the AO-driven H_2_O_2_ biosynthesis in the cell wall is well known to be involved in plant wound healing and pathogen attack responses by both triggering peroxidase-mediated wall-stiffening events and signaling modulation of defense gene expression. Extensive investigation by a variety of methodological approaches revealed high levels of expression of cell wall-localized AOs in root xylem tissues and vascular parenchyma of different plant species. Here, the recent progresses in understanding the role of cell wall-localized AOs as mediators of root xylem differentiation during development and/or under stress conditions are reviewed. A number of experimental pieces of evidence supports the involvement of apoplastic H_2_O_2_ derived from PA oxidation in xylem tissue maturation under stress-simulated conditions.

## 1. Introduction

In growing root tissues, meristematic cells deal with the need to coordinate sequential transitions from division to expansion and differentiation, which occur as separate phases at distinct developmental zones. In the Arabidopsis (*Arabidopsis thaliana*) root apex, four zones can be defined based on cell activity: (1) the meristematic zone up to 200 µm from the root cap; (2) the transition zone from about 200 up to 520 µm from the root cap; (3) the elongation zone from about 520 up to 850 µm from the root cap; and (4) the growth terminating zone from about 850 up to about 1500 µm from the root cap. In the latter zone, cell elongation slows down, and cells reach their final length [1]. In the proximal region beyond the zone of maximum elongation growth, protoxylem cells mature, undertaking the deposition of secondary walls [2]. The boundaries defining the division, elongation and maturation zones of the root are developmentally regulated. Of note, changes in their positions occur in a coordinated fashion during development, as reflected by the correlation observed in pea roots among root length, meristem size and protoxylem element position [3]. In Arabidopsis roots, the final meristem size is reached at five days post germination, when the rate of cell differentiation is balanced with the rate of cell division [4]. During development, the growth rate of the meristem is maintained stably during the plant’s lifespan by a complex cross-regulatory circuit relying on the antagonistic interaction between auxin and cytokinin, promoting respectively cell division in the proximal meristem and cell differentiation at the transition zone [4,5]. In this regard, it has been recently proposed that auxin defines the developmental zonation of division, expansion and differentiation activities by cooperating with the auxin-induced PLETHORA (PLT) transcription factor through different mechanisms [6]. Likewise, vascular patterning is specified through a mutually-inhibitory feedback between auxin and cytokinin, the former promoting and the latter inhibiting protoxylem identity [5,7,8]. However, the strong correlation among root length, meristem size and protoxylem element position observed in pea roots might cease under stress conditions, interfering with cell division, elongation or maturation events [3]. Of note, the stress signaling hormone jasmonic acid (JA) interferes with the auxin pathway involved in the maintenance of the root zonation by repressing PLT expression [9]. Furthermore, reactive oxygen species have also been involved in meristem size specification by controlling the transition between cell proliferation and differentiation, independently from the cytokinin/auxin pathway [10]. The present review illustrates recent insights into the role played in root development and xylem differentiation by hydrogen peroxide (H_2_O_2_) derived from the oxidation of polyamines (PAs) in the cell wall. In particular, we focused our attention on the involvement of cell wall-localized amine oxidases (AOs) in root xylem maturation under stress-simulated conditions.

## 2. Polyamines as Signaling Compounds and/or Hydrogen Peroxide Sources

### 2.1. Polyamines in Plants

PAs are low molecular weight aliphatic amines involved in various physiological and pathological events in plants, including growth, development, stress tolerance and defense responses [11,12,13]. The most common PAs are the diamine putrescine (Put), the triamine spermidine (Spd) and the tetramine spermine (Spm). Additionally, thermospermine (T-Spm), an isomer of Spm, which has not as yet been detected in mammalian cells, has been found to be widely distributed throughout the plant kingdom [14,15]. Put and Spd are essential for life, as Arabidopsis mutants defective in their biosynthetic pathways are embryo-lethal, whereas Spm and T-Spm have been specifically linked to stress responses and development, respectively [16,17]. Owing to the presence of regularly-spaced positive charges, PAs may act through stabilization of negatively-charged intracellular macromolecules, such as proteins, nucleic acids and phospholipids. However, besides their biophysical effects, these molecules may be involved in signal transduction pathways during developmentally-controlled programs or stress-induced responses and/or may exert their action as sources of biologically-active compounds, such as H_2_O_2_ [18,19]. In plants, a key signaling role in determining cell fate has been ascribed to the PA/H_2_O_2_ balance, especially in cell death associated with both defense and developmental processes [20,21].

### 2.2. Polyamine Signaling in Xylem Development

Alteration of PA homeostasis strongly affects higher plant architecture. An interplay between the PA and the cytokinin/auxin pathways has been revealed to occur in Arabidopsis. Perturbation of higher PA biosynthesis in the Arabidopsis loss-of-function *bud2* mutant, a knock-out of the *S*-*adenosylmethionine* *decarboxylase 4* (*SAMDC4*) gene necessary for Spd, Spm and T-Spm biosynthesis, results in a bushy and dwarf phenotype with altered vascularization due to an increase in the number of tracheary vessels along with a decrease in their size [22]. Considering that the *bud2* mutant displays hyposensitivity to auxin and hypersensitivity to cytokinin together with the findings that *BUD2* is inducible by the auxin signaling pathway, it has been hypothesized that PAs may affect plant architecture by both increasing sensitivity to auxin perception and repressing cytokinin biosynthesis and/or signaling [23]. Mutation of the *ACAULIS5* (*ACL5*) gene encoding the aminopropyl transferase driving T-Spm biosynthesis severely affects xylem specification in Arabidopsis hypocotyl. Bearing in mind that (1) *ACL5* is strongly expressed in provascular/procambial cells, (2) the *acl5* mutant displays over-proliferation and altered phenotype of xylem vessels, with the preponderance of very small and spiral-type xylem vessel elements and the absence of pitted vessels and xylem fibers, and (3) cell death occurs before the onset of secondary cell wall formation in *acl5* xylem vessels, a key role for T-Spm in preventing premature maturation and death of xylem elements has been suggested to allow complete expansion and correct secondary cell wall patterning [24,25,26]. It has also been proposed that T-Spm slows down xylem differentiation by antagonizing auxin signaling [14]. Indeed, in *acl5* seedlings, a number of genes related to auxin signaling have been shown to be upregulated, among which *MONOPTEROS* (*MP*) and its target genes, such as the *homeodomain-leucine zipper* (*HD-ZIP*) *III* encoding for the ARABIDOPSIS THALIANA HOMEOBOX8 (ATHB8) transcription factor. Of note, ATHB8 promotes the formation and differentiation of procambial cells into vascular cells [27,28,29]. An interplay between T-Spm and ATHB8 leading to a negative feedback loop has been supposed to occur in differentiating xylem cells as follows: in established xylem precursor cells in which patterning and differentiation triggered by auxin flow are taking place, ATHB8 induces the expression of *BUD2* and *ACL5*, leading to the biosynthesis of T-Spm, which, in turn, negatively affects the expression of *MP*, *HD-ZIP III* and key auxin signaling genes, thus counteracting the auxin-mediated differentiation of xylem precursor cells [27].

PAs may also play a role in the environmentally-induced plasticity of root architecture, by affecting primary root growth and lateral and adventitious root formation [30]. It has been suggested that exogenous Spd may act as a stress signal in maize (*Zea mays*) roots [31]. Indeed, coherent with the observation that stressed roots show a reduced rate of growth and altered architecture [30], exogenous Spd has been shown to inhibit primary root growth by affecting both the mitotic index and cell elongation in maize [32]. Furthermore, in Spd-treated maize roots, an earlier differentiation of xylem tissues has been revealed concurrently with an increase of cell wall phenolic autofluorescence in vascular tissues and rhizodermis [31], the latter event also being indicative of responses to stress, such as wounding in maize and tobacco (*Nicotiana tabacum*) [33,34]. Consistently, Spd supply has been shown to inhibit K^+^ uptake in maize roots, analogous to the effect of cutting roots into segments [35].

## 3. Terminal Polyamine Oxidation in the Cell Wall Is Triggered at Specific Developmental Stages or under Stress Conditions

### 3.1. Polyamines Are Oxidized by Copper and FAD-Dependent Amine Oxidases

**Figure 1:**
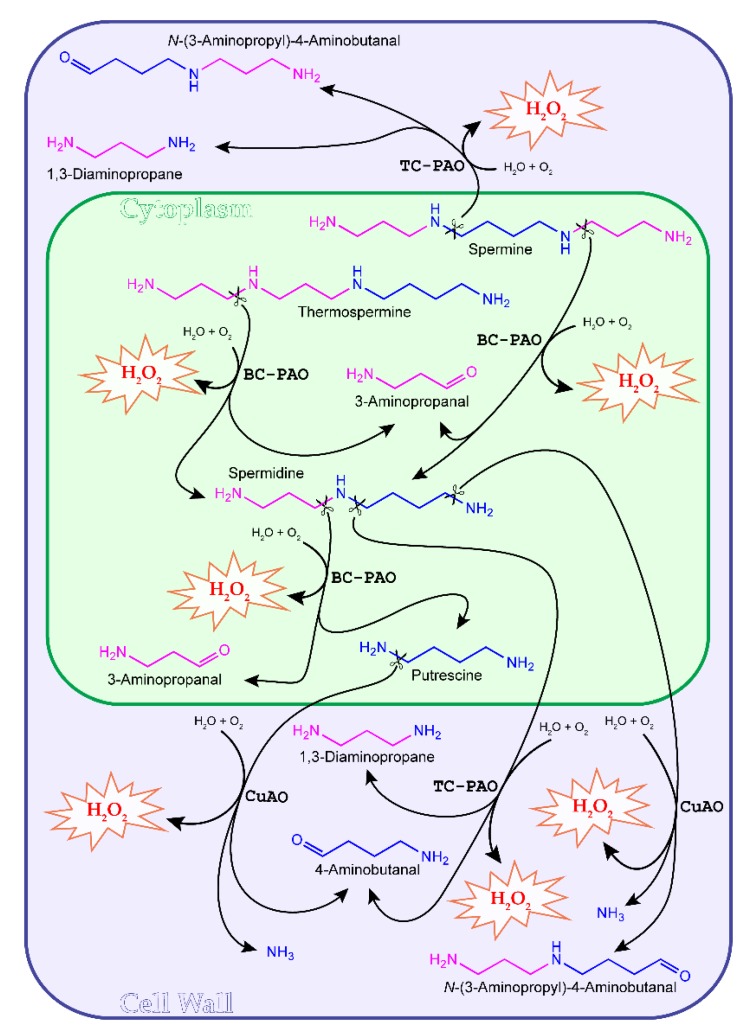
Polyamine (PA) oxidation in plants. The compartment with the violet background highlights PA terminal oxidations occurring in the cell wall. Scissors indicate cleavage sites. The peroxisomal Put and Spd oxidation and the extracellular monoamine oxidation carried out by *Arabidopsis thaliana* and *Malus domestica* CuAOs, as well as the vacuolar *Hordeum* *vulgare* HvPAO2-mediated oxidation of Spm and Spd are not shown for simplicity. TC, terminal catabolism; BC, back-conversion; PAO, polyamine oxidase.

AOs oxidize PAs to amino aldehydes, with the production of an amine moiety and H_2_O_2_ [15,19]. The reaction products vary depending on both the substrates and enzymes involved (Figure 1). The cell wall copper amine oxidases (CuAOs) purified from *Fabaceae* preferentially oxidize Put at the carbon next to the primary amino group with the production of 4-aminobutanal, ammonia and H_2_O_2_ (Figure 1) [36]. However, out of ten annotated *Arabidopsis thaliana* CuAOs (AtCuAOs), the apoplastic AtAO1 (At4g14940) and AtCuAO1 (At1g62810) and the peroxisomal AtCuAO2 (At1g31710) and AtCuAO3 (At2g42490) have been shown to additionally oxidize Spd at the primary amino group with an affinity comparable to that for Put, producing *N*-(3-aminopropyl)-4-aminobutanal, ammonia and H_2_O_2_ (Figure 1) [19,37,38,39,40,41].

On the other hand, a *Malus domestica* peroxisomal CuAO (MdAO1), highly expressed in fruits, predominantly oxidizes the diamines Put, cadaverine and 1,3-diaminopropane, but not Spd and Spm [42], while the extracellular MdAO2 represents the first plant CuAO so far described able to exclusively utilize long chain aliphatic and aromatic monoamines as substrates [42]. In tobacco, an apoplastic CuAO activity responsible for the extracellular oxidation of Put has been described [43], along with two peroxisome-resident CuAOs, the *N*-methylputrescine oxidase 1 (MPO1) and the *Nicotiana tabacum* diamine oxidase 1 (NtDAO1, formerly known as MPO2), respectively involved in nicotine biosynthesis and peroxisomal Put oxidation [39]. FAD-dependent polyamine oxidases (PAOs) oxidize Spd and Spm at the carbon located at the endo- or exo-side of the *N*^4^ atom by either a terminal catabolism (TC-PAOs) or a back-conversion (BC-PAOs) pathway, the latter being responsible for recycling Spd and Put, respectively from Spm and Spd. In particular, TC-PAOs oxidize the carbon at the endo-side of the *N*^4^ of Spd and Spm, producing 4-aminobutanal and *N*-(3-aminopropyl)-4-aminobutanal, respectively, in addition to 1,3-diaminopropane and H_2_O_2_, whereas BC-PAOs oxidize the carbon at the exo-side of the *N*^4^ of Spd and Spm to produce Put and Spd, respectively, in addition to 3-aminopropanal and H_2_O_2_ [15,19,44,45]. Some BC-PAOs from Arabidopsis and rice (*Oryza sativa*), including AtPAO1, OsPAO1, OsPAO4 and OsPAO5, are able to recycle Spd by oxidation of T-Spm, with the production of 3-aminopropanal and H_2_O_2_ (Figure 1) [19,44,45,46,47]. Furthermore, different from other TC- and BC-PAOs so far characterized, AtPAO5, which catalyzes the back-conversion of *N*^1^-acetyl-Spm, T-Spm and Spm to Spd, has been shown to have a 180-fold higher activity as a dehydrogenase than as an oxidase [48,49], thus suggesting that this enzyme does not have a role in H_2_O_2_ production, but rather plays a role in PA homeostasis. The TC-PAOs so far described in *Poaceae* and *Nicotiana tabacum* are secretory and mostly targeted to the cell wall, with only one exception represented by the barley (*Hordeum vulgare*) PAO isoform HvPAO2, which is targeted to the vacuole [15,19,43,50]. On the contrary, the BC-PAOs currently described in Arabidopsis and rice have an intracellular localization [44,46,47,48,49].

### 3.2. Features and Roles of Polyamine Oxidation in the Apoplast

A specific feature of apoplastic PA oxidation is the absence of the PA inter-conversion pathway in this compartment [44,48]. As a consequence, the availability of free soluble PAs in the cell wall would mainly depend on events of PA secretion, which may occur under environmental stresses or at specific developmental stages. Indeed, while in the absence of any internal or external stimulus, PAs are present in the cell wall at very low or even undetectable levels [51], under specific adverse conditions, such as pathogen infection or salt stress, they can be transported in the cell wall [20,43,52,53,54], thus becoming available for the apoplastic AOs. Likewise, AOs have been shown to undergo redistribution from cytoplasm towards the cell wall depending on developmentally-regulated or light-induced tissue maturation [55,56]. Hence, it can be stated that a complex interplay of events modulating both AO and PA levels and the rate of their secretion in the cell wall governs the spatio-temporal features of the AO-dependent biosynthesis of extracellular H_2_O_2_, which has been shown to play the dual role of triggering peroxidase-mediated wall stiffening events and signaling the modulation of defense and hypersensitive response (HR)-cell death gene expression [57,58].

Apoplastic CuAOs and TC-PAOs have been found at very high levels in several species belonging to *Fabaceae* and *Poaceae* families, respectively, especially in tissues fated to undertake extensive wall stiffening events, such as xylem, xylem parenchyma, endodermis and epidermis, and/or in cells undergoing programmed cell death (PCD), namely tracheary elements and root cap cells [57,59]. Consistently, in Arabidopsis, the cell wall-localized AtAO1 has been reported to be expressed in root cap cells and protoxylem precursors at early stages of vascular tissue differentiation [38,60].

In the cell wall, CuAOs and PAOs share overlapping roles as H_2_O_2_ sources in developmentally- or light-regulated cell wall maturation events [55,56,57,59], as well as in the oxidative bursts occurring during defense responses against biotic and abiotic stresses [57,61], especially during pathogen attack [16,19,38,43,54,57,62,63], salt stress and wound healing [20,33,52,57,61,64,65,66]. Consistently, the expression of cell wall-localized AOs is induced by stress signaling hormones. Indeed, the expression of TC-PAO from *Zea mays* (ZmPAO), CuAO from *Cicer arietinum*, *AtAO1* and *AtCuAO1* is strongly induced by JA and/or methyl-jasmonate (MeJA) treatments [33,40,60,64]. *AtCuAO1* expression is also induced by abscisic acid, salicylic acid and flagellin 22 treatments [40]. In recent years, growing evidence has suggested the involvement of H_2_O_2_ derived from the apoplastic AO-mediated PA-oxidation in root xylem differentiation under stress conditions, as discussed in detail in the following paragraphs [19,34,60,67].

## 4. Apoplastic Spermidine Oxidation Mediates Early Xylem Differentiation in the Maize Primary Root

In maize, three genes (*ZmPAO1*, *ZmPAO2* and *ZmPAO3*) have been identified as encoding identical secretory proteins, overall reported as ZmPAO, which are involved in the terminal catabolism of Spd and Spm in the cell wall [15]. ZmPAO has been detected mainly in epidermis, rhizodermis, endodermis and vascular tissues by histochemical and immunocytochemical techniques [55,56,57,58,59,60,61,62,63,64,65,66,67].

### 4.1. ZmPAO Expression and Sub-Cellular Distribution Are Developmentally Regulated in Maize Primary Root

In maize primary roots, ZmPAO expression and its subcellular localization are finely regulated during development [56]. It has been demonstrated that differentiating sub-apical tissues show higher ZmPAO levels as compared to mature basal tissues, independently of the root age [56]. Moreover, a subcellular redistribution from cytoplasm towards cell wall depending on tissue maturation stages has been revealed. In this regard, the percentage of insolubilized tightly wall-bound ZmPAO enzyme activity remarkably increases in mature basal root portion *vs.* differentiating sub-apical root tissues, especially in older as compared to younger seedlings [56]. A comprehensive analysis of ZmPAO expression has revealed that in apical meristematic and sub-apical differentiating root tissues, this protein is mostly present in the cytoplasm of early and late metaxylem precursors, regardless of the root age. In particular, the ZmPAO enzyme activity appears early in procambium and sloughed root cap cells and greatly increases in stelar tissues up to 1000 µm from the apical meristem [67]. In more mature tissues, an intense enrichment of ZmPAO has been reported to occur in the primary and secondary walls of early and late metaxylem precursors as compared to the apical zones. This difference is strengthened in an age-dependent manner, appearing higher in older roots [56].

Interestingly, an analogous sub-cellular redistribution of ZmPAO has been revealed in epidermal cells and xylem precursors of de-etiolated maize mesocotyls, as a consequence of light-induced tissues differentiation [55,56]. In detail, exposure of dark-grown maize seedlings to intense white light induces a marked increase of ZmPAO expression associated with its secretion in the cell wall [55], suggesting that under this condition, an enhanced catabolism of PAs in the apoplast may occur by modulation of both AO expression and secretion of the encoded proteins to the apoplast.

Overall, redistribution of ZmPAO from cytoplasm toward cell wall has been shown to be concomitant with tissue differentiation, such as during light-induced and developmentally-regulated maturation of mesocotyl and root tissues, respectively [55,56].

### 4.2. ZmPAO-Driven Oxidation of Spermidine in the Apoplast Mediates Early Xylem Differentiation in Maize Primary Root

Exogenous Spd affects maize root development by inhibiting elongation and altering cell cycle phase distribution in the root apex [32,67]. Concerning this, the key role in root growth inhibition and xylem differentiation of H_2_O_2_ derived from Spd oxidation (Spd-derived H_2_O_2_) has been demonstrated *in vivo* by exploiting a specific and selective inhibitor of the ZmPAO enzyme activity, as well as a H_2_O_2_ (*N*,*N*^1^-dimethylthiourea (DMTU)) trap, which allows fast removal of this compound soon after its production [67,68]. Furthermore, the increase in cell wall phenolic auto-fluorescence occurring in rhizodermis, xylem elements and vascular parenchyma has been shown to be mediated by the Spd-derived H_2_O_2_. Moreover, widespread in root tissues, Spd treatment induces nuclear condensation and DNA fragmentation, the latter being mediated by the Spd-derived H_2_O_2_. Likewise, precocious differentiation of early and late metaxylem occurring upon oxidation of exogenous Spd, whose position is closer to the tip compared to untreated roots, has been ascribed to the enhanced production of H_2_O_2_, which is clearly visible in xylem parenchyma and in differentiating xylem elements. However, the precocious cell death occurring in early differentiating xylem elements hinders full differentiation of the secondary wall [67]. The phenotype shown by Spd-treated roots is consistent with the hypothesis that exogenous PAs may contribute to create a stressing environment by simulating apoplastic accumulation of PAs reported to occur in stressed plants. Considering this, it could be reasonable to suppose that Spd-derived H_2_O_2_ mediates early xylem differentiation under stress conditions.

## 5. Cell Wall PAO and CuAO Signal Early Xylem Differentiation in Roots of Tobacco Plants

In tobacco, PAO and CuAO are highly expressed in cells destined to undergo lignification, especially in vascular tissues [69]. Both CuAO and PAO enzyme activities have been detected in the intercellular washing fluids of tobacco leaves [43]. These enzymes, which catalyze the terminal apoplastic oxidation of Put and Spm, are respectively inhibited *in vivo* by the well-known CuAO and PAO inhibitors, 2-bromoethylamine (2-BrEt) and *N*,*N′*-diaminoguanidine and guazatine [34,43].

### 5.1. Overexpression of ZmPAO in Tobacco Plants Induces Early Differentiation of Root Vascular Tissues

Ectopic overexpression of ZmPAO in the cell wall of tobacco plants drives early xylem differentiation associated with enhanced H_2_O_2_ production in root apex and induces PCD in root cap cells [67]. In these plants, oxidation of developmentally-secreted PAs may be accelerated by the overexpressed ZmPAO, simulating the high level of PA oxidation occurring in stressed plants and, thus, leading to an altered phenotype [67].

### 5.2. CuAO Mediates Early Xylem Differentiation in Transgenic Tobacco Plants with Constitutively-Activated Defense Responses

*Nicotiana tabacum* plants overexpressing a fungal endopolygalacturonase (PG plants) show constitutively-activated defense responses, owing to the production of an excess of oligogalacturonides (OGs) that are perceived by the plant as damage-associated molecular patterns [70]. H_2_O_2_ accumulation is a specific feature of PG plants [70], which also display higher CuAO activity and lower PA levels in leaves, especially Put, with respect to wild-type (WT) plants [34]. Histochemical analysis has shown a tissue-specific expression of CuAO in xylem cells, vascular cambium and neighboring derivative cells of petioles and stems of PG plants [34]. Moreover, phenotypic analysis of root apices from PG plants has revealed an early xylem differentiation associated with an enhanced accumulation of extracellular H_2_O_2_. Of note, in the roots of PG plants, Put level sharply decreases as compared to the value detected in roots of WT plants [34]. Overall, these results suggest that the root xylem phenotype could be associated with a perturbation of PA metabolism. The key role of the CuAO activity in early differentiation of root xylem precursors and extracellular H_2_O_2_ accumulation in PG plants has been established by exploiting *in vivo* the specific CuAO inhibitor 2-BrEt. Concerning this, upon 2-BrEt treatments, the position of xylem precursors with secondary cell wall thickenings partially recover to that observed in the WT, while inhibitor treatments are ineffective in WT roots. Furthermore, H_2_O_2_ accumulation detected in PG plants is strongly reversed by treatments with 2-BrEt. As a whole, these results suggest a role for the CuAO-driven Put oxidation in xylem differentiation under stress conditions, such as those signaled by pectin integrity alteration [34].

## 6. Hydrogen Peroxide Produced by the Apoplastic Copper Amine Oxidase 1 (*AtAO1*) Signals the Methyl-Jasmonate-Mediated Protoxylem Differentiation in Arabidopsis Roots

A comprehensive analysis of *AtAO1* expression pattern in Arabidopsis root apex has revealed a vascular tissue localization, especially in the transition, elongation and maturation zones, as well as in the root cap cells. Proceeding basipetally along the root length, from the transition zone up to the elongation and maturation zone, *AtAO1* promoter activity has been detected in protoxylem precursors, whole vascular cylinder and metaxylem precursors [60]. MeJA strongly induces *AtAO1* expression in the vascular cylinder, concurrently with a spatial anticipation of its pattern, which appears closer to the transition zone upon the hormone treatment. However, *ATAO1* loss-of-function mutants display no altered phenotype in root xylem tissues in comparison to WT plants [60], suggesting an irrelevant or non-prevalent role of *AtAO1* in vascular development under physiological growth conditions or, alternatively, the occurrence of a redundancy effect caused by another member of the CuAO gene family. Conversely, the evidence that MeJA treatments are effective in inducing early protoxylem differentiation in WT seedlings without affecting it in *AtAO1* mutants reveals the involvement of *AtAO1* in the MeJA-signaled differentiation of root vascular tissues. The hypothesis that MeJA’s effect on protoxylem differentiation can be mediated by the H_2_O_2_ produced via the *AtAO1*-driven Put oxidation is supported by: (1) the reduction of Put levels in roots of MeJA-treated WT plants; (2) the reversion effect exerted by DMTU on MeJA-induced early protoxylem differentiation; and (3) the increased accumulation of H_2_O_2_ upon the same treatment at the site of appearance of protoxylem cells with fully-developed secondary wall thickenings [60]. Of note, Put-derived H_2_O_2_ modulates the protoxylem position under MeJA-signaled stress conditions independently of changes in the whole root elongation rate and meristem size [60]. Furthermore, Put supply and *AtAO1* overexpression also induce early protoxylem differentiation [60]. Overall, these data suggest that *AtAO1*-driven production of H_2_O_2_ may play a role in xylem differentiation under stress conditions, such as those signaled by MeJA or simulated by either Put treatments or *AtAO1* overexpression.

## 7. Conclusions and Future Perspective

**Figure 2:**
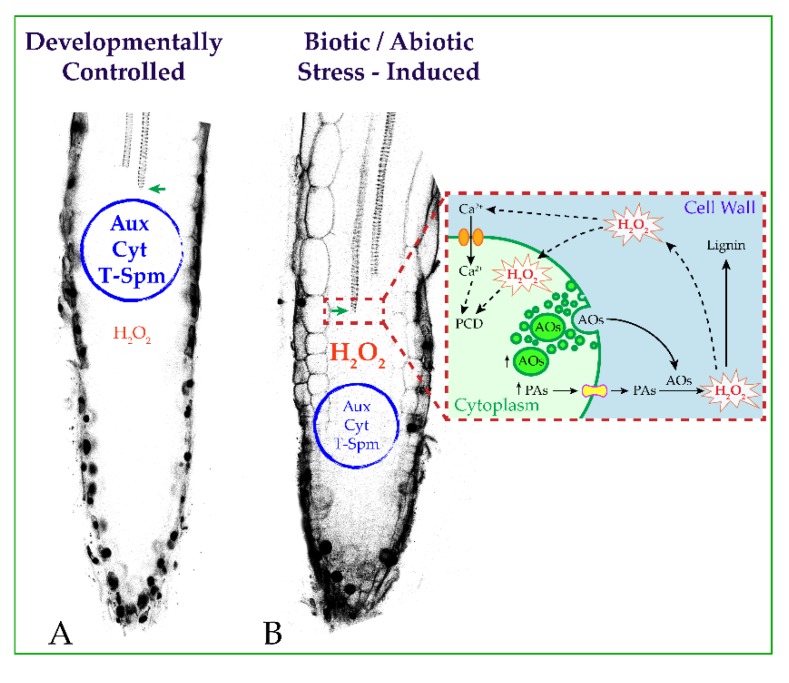
Schematic representation of the hypothetical stress-induced signaling triggered by PA oxidation and leading to root xylem differentiation. (**A**) Developmentally-controlled xylem differentiation is governed by the auxin/cytokinin/T-Spm loop. Apoplastic PA oxidation-derived H_2_O_2_ may contribute to the oxidative burst needed for full differentiation of the secondary wall. (**B**) Under stress conditions, an early root xylem differentiation occurs, and the first xylem precursor with secondary walls appears positioned closer to the root tip. This event is prevalently signaled by the H_2_O_2_ derived from the AO-driven terminal oxidation of PAs in the apoplast of differentiating xylem elements, independent of the auxin/cytokinin/T-Spm loop. (Inset in (**B**)) The square on the right illustrates a hypothetical scheme of the events at the level of differentiating xylem tissue under biotic/abiotic stress. Under these conditions, the expression of PA metabolic genes and apoplastic vascular-expressed AOs is induced along with PA and AO secretion in the cell wall. Terminal oxidation of PAs accumulated in the cell wall triggers an extracellular oxidative burst. H_2_O_2_ in the cell wall signals developmental cell death and acts as co-substrate in peroxidase-mediated lignin polymerization. PCD, programmed cell death.

In response to environmental stresses, plants may respond by accumulating PAs in the cell wall. Induction of apoplastic AO expression is also a feature of stressed plants. Considering this, treatments with exogenous PAs or apoplastic overexpression of AOs may simulate environmental stressing conditions by driving a PA-dependent oxidative burst in the cell wall. H_2_O_2_ derived from the AO-driven terminal oxidation of apoplastic PAs behaves as a co-substrate in the peroxidase-mediated wall stiffening events and as a signal for defense responses, such as HR-cell death and defense gene expression (Figure 2) [58].

Plants take advantage of the developmental plasticity of roots, whose architecture is deeply affected under changing environmental conditions. An early differentiation of xylem vessels has been revealed to occur in roots of plants under artificial stress conditions, such as those simulated upon exogenous PA treatments or apoplastic AO overexpression [60,67], or signaled by both MeJA treatment and pectin integrity alteration in transgenic PG plants [34,60]. The key role of the cell wall-localized AOs in the stress-induced xylem differentiation has been demonstrated by genetic and pharmacological approaches. Concerning this, PA-derived H_2_O_2_ has been shown to be involved as a mediator in the final events needed to accomplish full xylem differentiation, namely secondary wall deposition and developmental PCD (Figure 2). Overall, the reviewed evidence supports the hypothesis that PA oxidation mediated by apoplastic vascular-expressed AOs could assume a prevalent role in H_2_O_2_-mediated xylem differentiation under stress conditions, autonomously from the auxin/cytokinin/T-Spm loop.

In roots undertaking the early xylem differentiation process, in which fully-differentiated xylem elements appear positioned closer to the root tip, the stress-induced architecture of the water conducting system could be functional in improving the efficiency of water absorption under adverse conditions. The involvement of the apoplastic vascular-expressed AOs in the optimization of water uptake deserves to be carefully explored.
